# Cerebrospinal fluid solute transport associated with sensorimotor brain activity in rodents

**DOI:** 10.1038/s41598-023-43920-2

**Published:** 2023-10-09

**Authors:** Evgenii Kim, Jared Van Reet, Seung-Schik Yoo

**Affiliations:** Department of Radiology, Brigham and Women’s Hospital, Harvard Medical School, 75 Francis Street, Boston, MA 02115 USA

**Keywords:** Neuroscience, Physiology

## Abstract

Cerebrospinal fluid (CSF) is crucial for maintaining neuronal homeostasis, providing nutrition, and removing metabolic waste from the brain. However, the relationship between neuronal activity and CSF solute transport remains poorly understood. To investigate the effect of regional neuronal activity on CSF solute transport, Sprague–Dawley rats (all male, n = 30) under anesthesia received an intracisternal injection of a fluorescent tracer (Texas Red ovalbumin) and were subjected to unilateral electrical stimulation of a forelimb. Two groups (n = 10 each) underwent two different types of stimulation protocols for 90 min, one including intermittent 7.5-s resting periods and the other without rest. The control group was not stimulated. Compared to the control, the stimulation without resting periods led to increased transport across most of the cortical areas, including the ventricles. The group that received intermittent stimulation showed an elevated level of solute uptake in limited areas, i.e., near/within the ventricles and on the ventral brain surface. Interhemispheric differences in CSF solute transport were also found in the cortical regions that overlap with the forelimb sensorimotor area. These findings suggest that neuronal activity may trigger local and brain-wide increases in CSF solute transport, contributing to waste clearance.

## Introduction

The brain is a highly metabolic organ that produces a significant amount of waste byproducts from neuronal activity^1^. Thus, efficient clearance of waste from the brain is essential to maintain its normal homeostasis. Suboptimal waste clearance and aberrant brain lymphatic function have been observed in several pathological conditions, including stroke^2,3^, traumatic brain injury^4^, normal pressure hydrocephalus^5^, as well as physiological factors such as hypertension^6^ and aging^7,8^. Although the direct relationship between waste clearance and its buildup in the brain is still under intense investigation, progressive accumulation of metabolic byproducts and their derivatives, such as beta-amyloid and tau-protein plaques, is a hallmark sign in Alzheimer's disease^9^. Similarly, inefficient waste clearance from the brain is found to be associated with the accumulation of alpha-synuclein proteins in Parkinson's disease^10^ and dementia with Lewy bodies^11,12^.

The lymphatic system, other than its important function in mediating immune responses and fluid balance, plays a critical role in removing waste from organs^13^. Although the detailed cytoarchitecture and biophysical mechanism involved in the lymphatic function of the central nervous system (CNS) is not fully understood, it is known that the CNS lymphatic system is different in both anatomy and function from those found in other organs^14–16^. Studies have suggested that waste produced by neuronal cells, in the form of solutes, is transported to the interstitial fluid (ISF) and subsequently exchanged with the cerebrospinal fluid (CSF) via various mechanisms, including aquaporin-4 (AQP4) water channels^17,18^, osmotic pressure gradient^19^, active ion transport^20^, and passive diffusion^21^, before its ultimate exit from the CNS to the peripheral lymphatic system. Thus, CSF solute transport in the brain plays an important role in removing waste products from the brain^22,23^.

The presence and directionality of CSF movement around the cerebral vasculature have recently been identified by Mestre et al.^24^ whereby CSF solutes move along the perivascular space (PVS) which runs parallel to cerebral blood vessels, facilitating CSF/ISF solute exchange. The flow of CSF within the PVS has been shown to be driven by the contraction of smooth muscle cells surrounding the cerebral arterioles^25–28^. Meanwhile, neuronal activity is accompanied by localized vascular responses (i.e., hemodynamic responses), such as changes in local cerebral blood flow (CBF)/cerebral blood volume (CBV), which are mediated by vessel wall dilation/contraction^29^. Recent studies have demonstrated the potential of vagus nerve stimulation to modulate CSF flow in rats^30,31^. Moreover, the cerebral vasodilation triggered by whisker stimulation in sensory regions of mice has been accompanied by increased CSF influx velocity within the activated hemisphere^32^. Additionally, photic stimulation of the visual cortex in humans yielded large-scale CSF flow that is aligned with visually evoked hemodynamic responses^33^. Despite these recent studies of CSF dynamics that are associated with neuronal activity, evaluation of the location and the degree of CSF solute transport responding to region-specific brain stimulation warrants further investigation.

In the present study, we investigated the impact of region-specific neuronal activity within the sensorimotor cortex elicited by unilateral electrical stimulation of a forelimb on CSF solute transport in anesthetized Sprague–Dawley (SD) rats. The selection of forelimb stimulation mitigates potential confounding cardiovascular and respiratory effects on CSF solute transport associated with cranial nerve stimulation^30,31^. Furthermore, the unilateral sensorimotor stimulation scheme allows for interhemispheric comparison, unlike the bilateral photic stimulation employed in the study by Williams et al.^33^ We evaluated the effects from two stimulation conditions that differ in the number of stimulations and their timing (with or without an introduction of resting periods), which are known to contribute to different cerebral hemodynamic responses^34^. Herein, we refer to the sustained stimulation without the introduction of a time gap as the sequential condition ('SEQ'), and the stimulation that accompanied a 7.5 s resting period between the batch of stimulation as the intermittent condition ('INT'). The SEQ condition would lead to an increase in blood flow until reaching a static level^35^, while the 7.5 s resting between stimuli in the INT condition would yield periodic hemodynamic changes^36^, thereby inducing dynamic vascular responses. To assess CSF solute transport impacted by the stimulation, we adopted a well-established technique involving the intracisternal injection of a fluorescent CSF tracer^4,6,37^. The tracer injection was conducted prior to initiating the stimulation, after which we evaluated the distribution of the tracer on the dorsal brain surface and within coronal sections that encompassed the stimulated brain regions.

## Material and methods

### Animals

All animal procedures were conducted in accordance with the guidelines of the Care and Use of Laboratory Animals and were approved by the Institutional Animal Care and Use Committee (IACUC) of Brigham and Women's Hospital (protocol 2020N000013). The reporting in this study is in accordance with the ARRIVE guidelines (https://arriveguidelines.org/). Eight week-old male SD rats (280.6 ± 10.9 g, n = 30, Charles River Laboratories, Wilmington, MA) were socially housed (two per cage) and maintained on a 12 h/12 h light/dark cycle (lights on at 7 AM, off at 7 PM) with ad libitum access to food and water.

### Intracisternal CSF tracer injection

The rats were anesthetized using intraperitoneal (*i.p.*) injection of a ketamine-xylazine mixture (80:10 mg/kg). The choice of ketamine-based anesthesia was based on prior research demonstrating a minimal effect of ketamine on sensory evoked potentials^38^. The depth of anesthesia was determined by the absence of pedal reflex to a toe pinch, and additional doses of anesthesia were administered as needed. After removing the fur over the scalp using clippers and depilation lotion, the animals were placed on a stereotaxic frame (ASI, Warren, MI, USA) after the application of ophthalmic ointment to the eyes, with the head tilted down ~ 30° ventrally for surgical access to the cisterna magna. The CSF tracer was administered via intracisternal injection using a previously established surgical protocol^39^. Briefly, a midline incision was made between the ears along the neck. Subcutaneous tissues and posterior vertebral muscles were retracted to expose the atlantooccipital membrane, and a 30-gauge needle filled with sterile artificial CSF (aCSF, Tocris, Fisher Scientific, Hampton, NH) was inserted into the cisterna magna space at a depth of ~ 1–2 mm. Cyanoacrylate glue (454 Loctite, Henkel, Düsseldorf, Germany) was applied around the injection site to seal the puncture while securing the needle in place. Texas-Red OA (023,021, Thermo Fisher, Waltham, MA) in aCSF (0.5 wt% concentration) was loaded in a polyethylene tube (0.28 mm inner diameter, 427,400, Becton Dickinson, Sparks, MD) and delivered into the CSF space at a rate of 2 µL/min for 10 min using a syringe pump (Legato 100, KD Scientific, Holliston, MA). Following tracer administration, the open end of the tube was sealed to prevent backflow of the tracer solution, and the animal's head was returned to its natural prone position. The body temperature of the animals was maintained throughout the procedure using a water-circulating pad (36 ºC, T-pump, Gaymar, Orchard Park, NY).

### Forelimb sensorimotor stimulation

**Figure 1:**
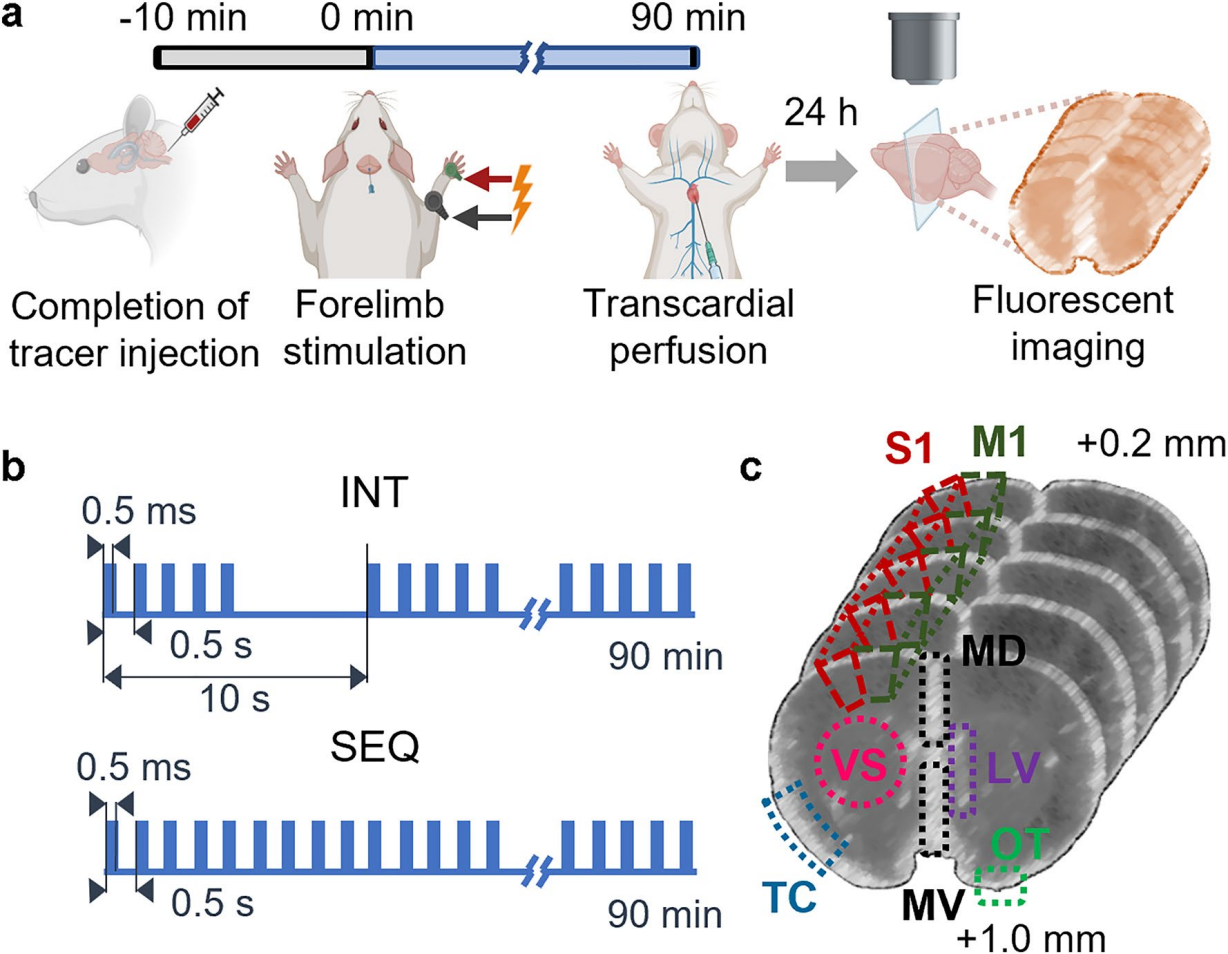
Schematic diagram of the experimental procedure, electrical stimulation, and ROIs. (**a**) Intracisternal injection of the fluorescent CSF tracer was followed by electrical stimulation of either the right or left forelimb (side randomized and balanced). Transcardial perfusion was performed immediately after stimulation, and brain imaging was performed after 24 h. (**b**) Stimulation parameters for two distinct protocols, labeled as 'INT' and 'SEQ'. (**c**) Location of designated ROIs on coronal brain slices, including the medial ventral (MV) and dorsal (MD) regions, primary motor (M1) and sensory (S1) cortices, temporal cortex (TC), olfactory tubercle (OT), ventromedial striatum (VS), and lateral ventricle (LV). Figure created with BioRender.com.

Overall experimental procedure is illustrated in Fig. 1a. Ten minutes after the completion of tracer administration, a unilateral electrical stimulation was applied to the forelimb of the rats, with the side of stimulation randomized and balanced across the animals. Animals were randomly assigned to one of three experimental conditions (Fig. 1b) (n = 10 for each group): (1) an intermittent stimulation (INT), (2) a sequential stimulation (SEQ), or (3) no stimulation as a control. Both stimulation protocols were composed of short 0.5 ms pulse durations and a repetition rate of 2 Hz, which are parameters previously utilized in preclinical and clinical research^40,41^. The INT condition entailed the delivery of a train of five electrical pulses (0.5 ms pulse duration) at a frequency of 2 Hz, with 10 s intervals between initiation of each pulse train, for a duration of 90 min. The SEQ condition delivered electrical pulses with the same pulse durations and frequency (in 90 min), but without introducing the intervals in-between. Two electrical stimulation conditions were used to assess the effects of non-stimulated resting period (i.e., 7.5 s) on the CSF solute transport. The choice of a relatively low stimulation frequency (2 Hz) was intended to mitigate the risk of neuronal adaptation during sustained stimulation (for 90 min) during the SEQ condition, while producing robust evoked responses^42,43^.

The electrical stimulation was delivered through a pair of cuff electrodes, with the positive wire above the manus and the negative wire wrapped beneath the cubitus, using a stimulator (ML260, ADInstruments, Australia). The stimulation intensity for each rat was individually adjusted between 7 and 14 mA until paw muscle twitching was observed. The rats in the control group underwent the same surgical and stimulation preparations but without the application of electrical stimulation. To evaluate whether the CSF solute transport was affected by heart rate or anesthetic depth^44^, we monitored heart rate and oxygen saturation (SpO_2_) every 5 min during the stimulation period using a pulse oximeter (Surgivet Hand-held V1030, Smiths Medical, Dublin OH). The respiration rates were counted manually.

### Brain harvesting and fluorescence imaging

Animals were sacrificed immediately after the electrical stimulation through transcardial perfusion of 150 mL normal saline followed by 150 mL of 4% paraformaldehyde solution (PFA in phosphate-buffered saline) at a flow rate of 25 mL/min. The skulls were detached then immersion-fixed for an additional 24 h in PFA prior to brain harvesting. After harvesting the brain, the dorsal surface of the brain was imaged using an inverted fluorescent microscope (TS100, Nikon) equipped with an ultrawide field CMOS sensor (23.4 × 15.6 mm, NEX-5, Sony) at an image size of 4912 × 2760 pixels. Bright-field and fluorescence images were acquired using a broadband light source and a fluorescent light configuration (EM: 560 nm/EX: 620 nm), respectively. Then, a block encompassing ± 2 mm (rostral and caudal) from bregma was then cut and embedded in low gelling-temperature agarose (A4018, Millipore Sigma, Burlington, MA; 3% weight in phosphate-buffered saline) and sectioned using a vibratome (PELCO easiSlicer, Ted Pella, Redding, CA) in the rostral-caudal direction from + 1 to + 0.2 mm (from bregma) in 200 µm-thick slices, yielding five slices that contain the brain regions associated with forelimb stimulation^45^. Both bright-field and fluorescence images were taken under a fixed exposure time (0.1 s and 1 s), respectively.

### Image analysis

The transport of CSF solutes was quantified from both surface and sectional fluorescent brain images after extracting red fluorescence using ImageJ software^46^. The dorsal surface fluorescent images from each animal were aligned together based on the corresponding bright-field images, guided by the rostral edge of the neocortex and the interhemispheric fissure as references. After alignment, the images were smoothed with a 2D Gaussian filter (standard deviation of 2) and down-sampled (492 × 276 pixels) to reduce the contribution of cortical edges associated with cerebral blood vessels to allow for group-averaging of the fluorescence images.

To evaluate the effects of neuronal activity on CSF solute transport within the sectioned brain block, mean pixel intensity (MPI) was calculated from fluorescent images across the five sectioned brain slices per animal. Region-specific solute transport was measured by separately calculating MPI from 14 regions-of-interest (ROIs) manually segmented from a co-registered rat brain atlas in relation to the corresponding sectional images^47^ (Fig. 1c). Two medial ROIs were: (1) the medial ventral (MV) area that includes the medial septal nucleus and the nuclei of the vertical/horizontal limb of the diagonal bands, and (2) the medial dorsal (MD) area that included the indusium griseum and interhemispheric fissure, all in the middle of the sectioned slice. The remaining bi-hemispheric ROIs consisted of (3) the ventromedial striatum (VS), (4) primary motor cortex (M1), (5) the forelimb sensory cortex (S1), (6) olfactory tubercle (OT), (7) lateral ventricle (LV), and (8) temporal cortex (TC; containing the secondary somatosensory cortex, glomerular layer of the olfactory bulb, dysgranular insular cortex, posterior part of the agranular insular cortex, and piriform cortex). These bi-hemispheric ROIs were defined each from the side ipsi—(IL) and contra-lateral (CL) to the stimulated forelimb. We note that the use of IL and CL nomenclature was referenced to the side of the stimulated forelimb (rather than the brain hemisphere) as neuronal somatosensory activity occurs bilaterally due to interhemispheric connections^48,49^.

### Statistical analysis

Statistical analysis was conducted using Matlab software (Ver. 2022b, Statistics and Machine Learning Toolbox, MathWorks, Natick, MA). The differences in vital signs (heart rate, respiratory rate, and SpO_2_) across experimental conditions over the 90 min stimulation period were evaluated using multi-way ANOVA. One-way ANOVA was used to assess the difference in animal weights across groups and to compare the fluorescent intensity values (MPI) obtained from the brain block as well as from the individual ROIs across experimental conditions. Differences in solute uptake among groups were examined using a Fisher's least significant difference (LSD) *post-hoc* test. A one-tailed paired t test was used for pixel-wise comparison of fluorescence intensity value obtained from dorsal surface images within each group. The significance level was set at *p* < 0.05 except for the pixel-by-pixel analysis of the fluorescence from the dorsal brain surface (*p* < 0.01).

## Results

### Evaluation of weight, respiratory, heart rate, and SpO_2_

There was no difference in body weight among the three groups (Ctrl: 279.7 ± 7.4 g; INT: 281.6 ± 15.6 g; SEQ: 280.6 ± 9.2 g; one-way ANOVA, F(2,27) = 0.07, *p* = 0.93). The vital signs (respiratory/heart rates and SpO_2_) measured during the stimulation session are shown in Table 1. The measured vital signs remained in the normal range for the anesthesia and were not different across the experimental groups throughout the entire stimulation period (multi-way ANOVA, all *p* > 0.7).

**Table 1:** Respiratory/heart rates (RR and HR), and SpO_2_, measured every 30 min during electrical forepaw stimulation. The presence of potential changes in the RR, HR, and SpO_2_ within the group was evaluated using multi-way ANOVA.

		RR *(Breaths per minute)*				HR *(Beats per minute)*				SpO_2_ *(%)*			
	Time (min)	0	30	60	90	0	30	60	90	0	30	60	90
Ctrl	Mean	66	62.8	61.6	62	239	247	243.6	239.8	90.9	91.3	91.1	90.9
	Std	6.5	5.3	4.7	3.9	22.7	31.9	19.2	18	1.7	1.3	1.8	1.6
INT	Mean	66.8	61.2	61.2	61.2	242	239	241.6	242.8	90.8	91.7	91	90.1
	Std	6.2	8	4.6	6.1	29.2	41.3	30	18.1	2.5	2	2.3	2.7
SEQ	Mean	67.2	62	61	60.1	240	242.5	239.6	238.7	90.7	91.1	91.6	91.9
	Std	8.1	11.2	5.7	6.8	36.1	44.5	33.3	44.3	3	2.9	2.6	2.4
Multi-way ANOVA	F(2,114)	0.08				0.36				0.04			
	*P*	0.92				0.70				0.96			

### Total OA uptake in sectioned brain slices

**Figure 2:**
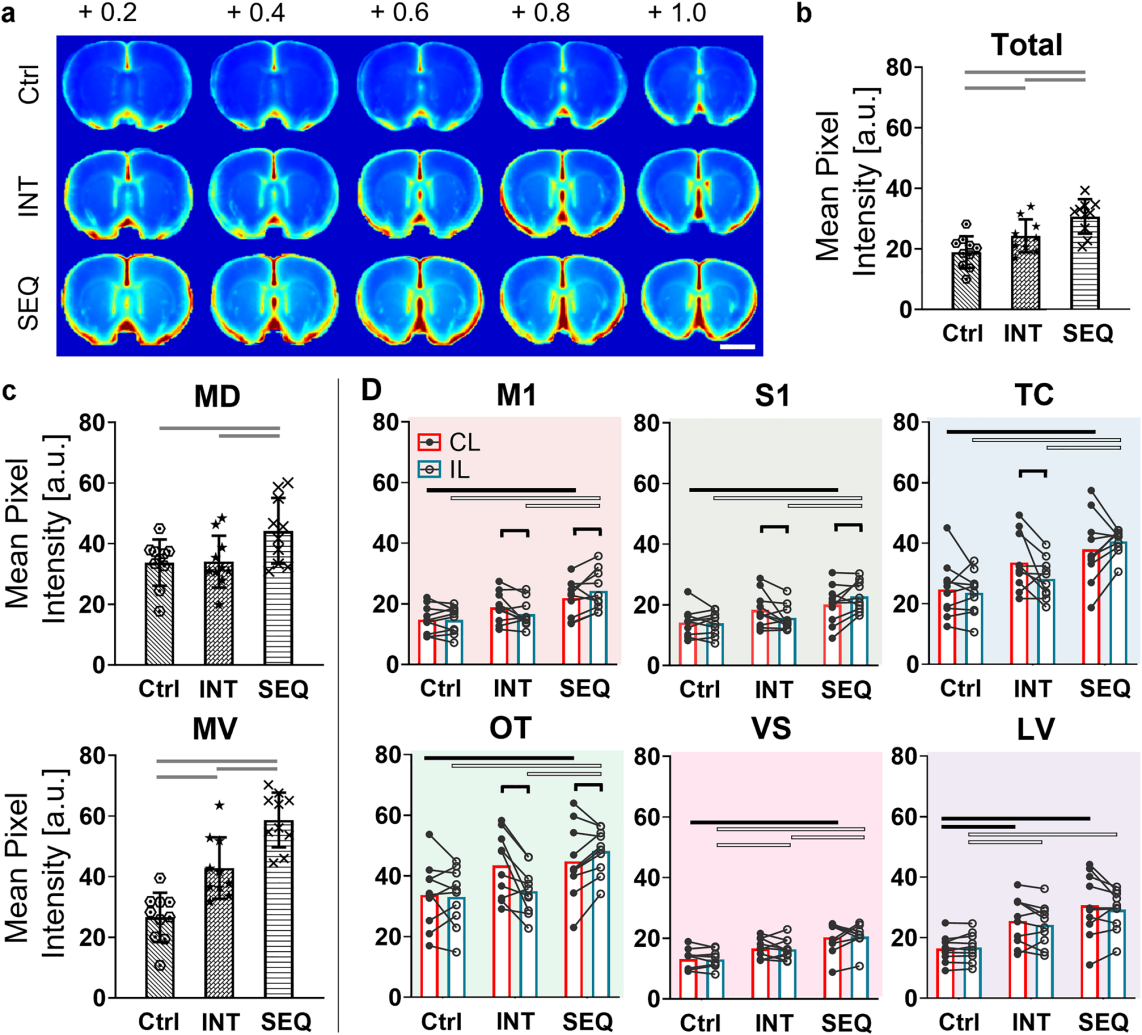
Cerebrospinal fluid (CSF) solute transport associated with neuronal activity. (**a**) Pseudo-colored group-averaged fluorescent images from the brain slices (from + 0.2 to + 1.0 mm from the bregma) across three experimental conditions. Bar = 5 mm. (**b**) The condition-specific mean fluorescent pixel intensity (MPI) calculated from a total area of the brain slices and (**c**) from medial dorsal (MD) and medial ventral (MV). Individual data are represented by symbols (hexagon, star, cross, dot). The error bars: standard deviation. The horizontal lines indicate a significant difference between experimental conditions (one-way ANOVA followed by Fisher's Least Significant Difference *post-hoc* test, p < 0.05). (D) The mean MPI values were obtained from bi-hemispheric ROIs (red bar: from the contralateral (CL) to limb stimulation, blue bar: from the ipsilateral (IL) side), with individual data shown in black and white dots, respectively. The horizontal lines indicate a significant difference across experimental conditions from the ROIs CL to stimulation (black line) and from the ROIs IL to stimulation (white line, *p* < 0.05 from one-way ANOVA followed by Fisher's Least Significant Difference *post-hoc* test). The brackets indicate a significant difference in MPI values between the CL and IL (one-tailed, paired t test, *p* < 0.05).

The aligned fluorescence images of brain slices were averaged within each condition to represent the group-level distribution of CSF solute transport from the brain block (Fig. 2a). One-way ANOVA analysis of MPI values reflecting a total area of the brain slices (Fig. 2b) showed a significant difference in solute uptake across the groups (F(2,27) = 11.7, *p* < 0.001), suggesting both SEQ and INT conditions yielded a higher total CSF solute uptake compared to the control. A *post-hoc* LSD analysis showed that the SEQ condition resulted in significantly higher MPI (30.7 ± 5.4) than the INT by 26.3% (24.3 ± 5.1; *p* < 0.05) and the control condition by 61.5% (19.0 ± 4.9; *p* < 0.001). Additionally, the INT condition exhibited a significantly higher MPI than the control condition by 27.9% (*p* < 0.05).

### ROI analysis on the OA uptake

The individual MPI values obtained from each ROI are reported in Supplementary Table S1–S3. The MPI values from medial ROIs (MD and MV, Fig. 2c) were significantly different across the three experimental conditions (one-way ANOVA, both *p* < 0.05, detailed statistical results are presented in Supplementary Table S4). The LSD *post-hoc* analysis showed that the SEQ condition showed the highest fluorescence value in both medial areas compared to the INT and the control conditions (LSD *post-hoc,* both *p* < 0.05). The comparison of solute transport between the INT and control groups revealed higher MPI values in the INT from the MV area (LSD *post-hoc, p* < 0.05), but no difference was found in the MD (LSD *post-hoc, p* = 0.94).

In addition to these medial brain regions, all bi-hemispheric ROIs (Fig. 2d) exhibited a higher CSF solute transport in animals subjected to the SEQ condition than the control group (LSD *post-hoc* test following one-way ANOVA, *p* < 0.05; detailed statistical results are reported in Supplementary Table S4). When the solute uptake was compared between the INT and control conditions, a significant difference was observed from the VS contralateral to limb stimulation and bilateral LV (LSD *post-hoc*, all *p* < 0.05), while the marginal difference was observed in ipsilateral VS, TC, and OT regions (LSD *post-hoc*, all *p* = 0.06, Supplementary Table S4). Between SEQ and INT, SEQ condition resulted in higher solute uptake in the VS bilaterally and in M1, S1, TC, and TU ipsilaterally (LSD *post-hoc*, all *p* < 0.05).

### Hemispheric comparisons on the OA uptake among ROIs

When the MPI values from these ROIs were compared between the hemispheres (CL vs IL) within each group (significant difference shown in brackets, Fig. 2c), INT yielded a higher OA uptake in M1, S1, TC, and OT contralateral to stimulation than ipsilateral ROIs (one-tailed, paired t test, all *p* < 0.05). On the other hand, the SEQ showed lower fluorescent intensity in the contralateral M1, S1, and OT compared to the opposite hemisphere (one-tailed, paired t test, all *p* < 0.05). The control condition showed no significant interhemispheric differences.

**Figure 3:**
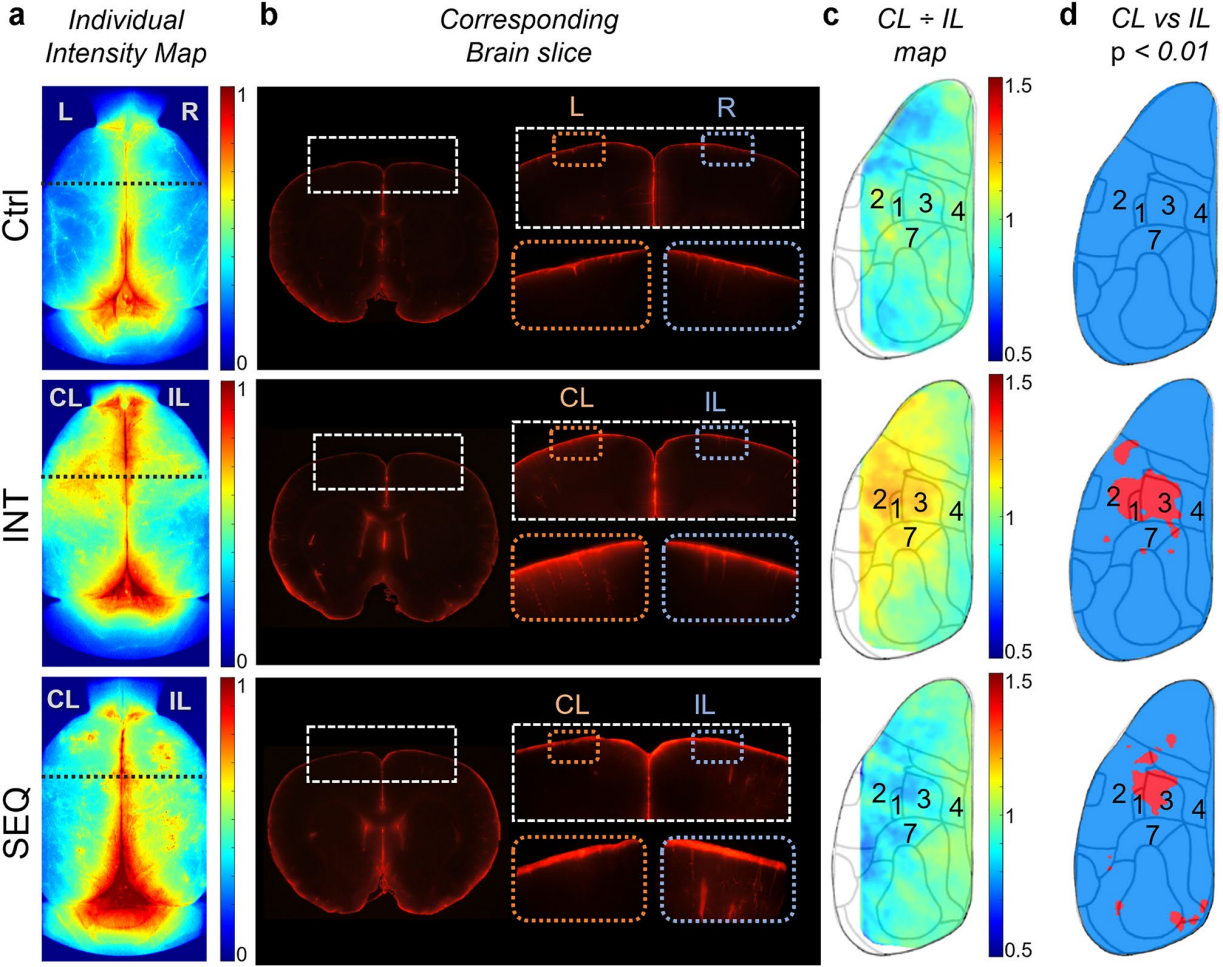
Interhemispheric difference in CSF solute transport associated with neuronal activity. (**a**) Representative fluorescent images of the brain surface of an animal from each experimental condition. 'CL': contralateral to electrical stimulation of a forepaw, 'IL': ipsilateral to the stimulation. In the control condition, the left and right side of the brain was shown due to the absence of electrical stimulation. (**b**) Sectioned brain slice (AP: + 0.6) from the location marked with black dotted lines from (**a**). The upper right insets are magnified from a region that encompasses the S1 and M1 (dashed box). The lower right insets are further magnified images from the cortical areas shown in orange (CL) and blue dotted boxes (IL). (**c**) The group-averaged fluorescence of the contralateral side normalized with respect to the ipsilateral hemisphere. (**d**) The cortical regions with a statistically significant difference (one-tailed paired t test, *p* < 0.01), being overlaid on the corresponding Brodmann areas, according to Krieg^37^.

Fluorescence images of the dorsal cortical surface across different experimental conditions (pixel values normalized to the maximum, Fig. 3a) showed asymmetrical OA transport resulting from the INT and SEQ conditions. The image along the coronal section of the forepaw sensorimotor areas (coordinate AP: + 0.6 mm; dotted line in Fig. 3a) showed that the higher fluorescence over the dorsal surface also extended into a deeper cortical area (Fig. 3b). The group-averaged ratio of pixel intensity from the brain surface between CL and IL sides confirmed the presence of asymmetric OA uptake between the hemispheres (> 1 indicates higher uptake in CL and < 1 indicates higher uptake in IL; Fig. 3c) while these regions shared spatial similarity to the functional areas associated with forelimb areas identified by direct electrical stimulation^45,50^ (listed by Brodmann's area nomenclature). Pixel-wise comparison between hemispheres, conducted via one-tailed paired t test, revealed areas that showed distinctive hemispheric differences (*p* < 0.01, Fig. 3d). On the contrary, the hemispheric differences in OA uptake on the brain surface were not observed in the control condition.

## Discussion

Despite extensive research, the exact routes of waste clearance from the brain are still unknown. However, increasing evidence suggests the importance of solute transport through the CSF in the clearance of waste produced by neuronal activity (e.g., beta-amyloid^51^ and lactate^52^). In the present study, we examined the effect of region-specific brain activity elicited by electrical forelimb stimulation on the transport of an intracisternally injected CSF tracer, Texas Red OA, in rats.

To determine the dependence of CSF solute transport on stimulation time scheme, two stimulation schemes (with and without an intermittent 'resting' period) were applied. Group-averaged fluorescence images of the coronal brain sections showed that neuronal activity increased CSF solute transport, and the overall OA uptake was higher in both the SEQ and INT stimulated groups compared to the unstimulated control condition (Fig. 2a). The results agree with a putative 'perivascular pumping' mechanism whereby increased neuronal activity and the accompanying increase in local CBF and CBV may enhance CSF solute transport^53–55^. The degree of the enhancement, as measured by MPI, was dependent on the stimulation scheme whereby the SEQ condition resulted in higher uptake than the INT condition (Fig. 2b). As to the causes for this observation, we postulate that the greater number of neuronal events per given time (i.e., 4 × greater) in the SEQ condition increased the neurovascular responses, which further promoted solute transport by CSF.

Heart rates and anesthesia, which are known to affect CSF solute transport^44,56,57^, were not different across experimental conditions, suggesting that their contributions to the enhanced CSF solute transport are not likely. We note that several physiological parameters known to impact CSF flow, such as intracranial pressure (ICP)^58^ and CO_2_ level^59^, were not directly monitored in our study as the procedure would involve invasive means to implement sensors. A study conducted by Illif et al. showed that ICP during intracisternal CSF tracer infusion in mice (1 µL/min for 10 min) resulted in a transient, mild elevation of (~ 2.5 mmHg) of ICP that rapidly recovered back to the pre-infusion level upon cessation^25^. A slightly higher infusion rate of 2 µL/min in our study, which was aimed to introduce enough tracer into a rat brain having much greater volume (by approximately four times), is unlikely to induce significant changes in ICP. It is supported by a study where a 60-min intracisternal infusion of a fluorescent tracer at a rate of 1.6 µL/min in rats did not perturb ICP^60^. The measured respiration rate and SpO_2_, which are closely linked to vascular CO_2_ level^61^, remained within the normal physiological range (60–67 breaths per minute and 90.1–91.9%; Table 1). Together, we surmise that the effects from potential alterations of ICP and vascular CO_2_ were negligible in our study. However, future research will benefit from controlled anesthetic protocols coupled with in vivo monitoring of ICP and vascular CO_2_ levels to ensure an unequivocal interpretation of the findings.

The regional assessment of OA uptake in the brain, represented in MPI values, revealed increased CSF solute transport in the medial regions of the brain (MD and MV) under the SEQ condition compared to the control condition, while animals subjected to INT stimulation showed higher OA uptake in the MV (Fig. 2c). Based on the group comparison of OA uptake from bi-hemispheric ROIs (Fig. 2d), although the electrical stimulation was given unilaterally, we found that both the SEQ and INT conditions showed an elevated level of CSF solute transport in both hemispheres compared to the control condition. Based on the premise of a correlation between neuronal activity and the degree of CSF solute transport, this bilateral enhancement seen from unilateral limb stimulation may be attributed to bilateral activation of a brain region via extensive interhemispheric connection^62–64^.

Furthermore, ROI-based analysis of MPI demonstrated that the SEQ stimulation led to increased transport of CSF solutes across all identified ROIs when compared to the control condition. This suggests that localized neuronal activity can cause changes in CSF solute transport at a macroscopic scale beyond the primary site of the neuronal activity. Animals under the INT condition, however, showed limited locations of ROIs with a significant increase in OA uptake compared to the control condition (i.e., the area proximal to and within the ventricles (i.e., VS and LV), medial ventral (MV) region, as well as the temporal cortex (TC)/olfactory tubercles (OT) contralateral (CL) to the limb stimulation). Although not statistically significant, the INT condition still showed a degree of elevation of OA uptake in most of the ROIs. As to the mechanism behind this observation, we hypothesize that fewer neuronal events (with the introduction of wait time of 7.5 s) during the INT condition compared to SEQ resulted in a lower degree of neurovascular response, which caused CSF solutes not being able to reach all ROIs under given observation time (90 min). We observed that the regions that showed a marginal elevation of solute uptake from the INT condition (compared to control) aligned with the known pathway of intracisternally injected tracers either along the ventricular compartment or along the ventral surface of the brain that connected to PVS adjacent to the middle cerebral arteries (illustrated in Supplementary Fig. S1 based on^65,66^). We conjecture that transport of intracisternally injected OA, following established major routes of CSF (i.e., ventral and via ventricular) was expedited in the SEQ condition compared to INT, resulting in the observed widespread solute uptake across the brain areas.

Interhemispheric differences in CSF solute transport following unilateral limb stimulation were found from the bilateral ROIs (Fig. 2d). Despite the presence of elevated levels of solute uptake in both SEQ and INT conditions, opposite trends in term of hemispheric OA uptake were observed. Specifically, the INT stimulation yielded higher OA uptake in the M1, S1, TC, and TO regions, contralateral to limb stimulation, compared to the ipsilateral hemisphere. On the other hand, the SEQ condition showed lower OA uptake in the M1, S1, and TC regions, contralateral to limb stimulation. These unequal interhemispheric uptakes of CSF solute with a bias toward the contralateral hemisphere during INT could be attributed to the predominantly activated neurons in the hemisphere contralateral to stimulation, which are accompanied by a higher CBF/CBV. Whereas the observed decrease in OA uptake within the stimulated hemisphere, as compared to the opposite side, during SEQ might be associated with a decline in neuronal activity resulting from prolonged stimulation at 2 Hz over a duration of 90 min. This notion aligns with a prior study demonstrating the initiation of reduction in neuronal response within the rodent primary somatosensory cortex following repetitive whisker stimulation at 2 Hz for 3 s^43^. Furthermore, repetitive sensory stimulation may lead to the release of inhibitory transmitters (e.g., adenosine, GABA) from activated astrocytes, resulting in a subsequent reduction of neuronal activity^67,68^. We also note that contribution from potentially persistent vasodilation^69^ or enlargement of cell volume^70^ might have played a role in impeding CSF movement within the PVS or interstitial solute transport during sustained neuronal stimulation during SEQ, subsequently reducing the solute transport efficiency^14,71^. Although our study primarily focused on the macroscopic distribution of CSF solute, in-vivo imaging techniques such as multi-photon microscopy^24^ may provide more detailed information about the tracer's movement and distribution in the PVS.

Fluorescence images of the dorsal brain surface also revealed a difference in OA uptake between hemispheres (Fig. 3), supporting the results from the ROI-based analyses. During INT, the contralateral hemisphere showed higher solute uptake than the ipsilateral hemisphere, whereas during SEQ, the contralateral hemisphere showed lower solute uptake. Pixel-by-pixel comparison of the MPI values between hemispheres revealed the brain regions having differential solute uptake overlapped with functional areas related to forelimb stimulation. This finding suggests that CSF solute transport has spatial specificity with respect to neural activity, although further validation through neuroimaging techniques such as functional magnetic resonance imaging or c-fos immunohistochemical staining is desired^72^.

The findings of this study are consistent with previous observations on the interplay between neuronal activity and CSF solute transport^30–33^, revealing the enhanced and region-specific solute transport associated with neuronal activity in anesthetized animals. It should be pointed out that previous studies on rodents have demonstrated that the state of brain activity, whether in an awakened or anesthetized state, significantly influences CSF solute transport^44,73^. Functional neuroimaging studies have further revealed spatial differences in neuronal response depending on the state of brain activity, with a higher spatial specificity during wakefulness in contrast to the widespread distribution observed under anesthesia^42,74^. Recent literature focusing on whisker stimulation also accentuated the presence of elevated CSF flow within the stimulated brain region under anesthetized conditions but not in awake mice^32^. Therefore, the possible effects of anesthetic agents on neuronal evoked potentials^75^ and CSF dynamics require further investigation in awake and natural sleep states.

Further direction for the current research may involve a more comprehensive investigation into diverse stimulation paradigms. The present study has demonstrated a significant difference in CSF solute transport depending on the hemodynamic responses induced by stimulation protocols, with and without the inclusion of intermittent resting intervals. However, it is important to note that various stimulation parameters such as intensity, rate, and duty cycle can exert a nonlinear impact on hemodynamic responses^76,77^. A prior study that engaged an intermittent sensory stimulation with a higher-frequency (5 Hz)^32^ showed an increase in CSF solute transport only in the stimulated hemisphere, where our results demonstrated that low-frequency stimulation influences CSF solute transport in both the stimulated and opposite hemispheres. This variation might be attributed to the unilateral 2 Hz stimulation evoking bilateral neuronal activation^42^. We also note that stimulation of a specific nerve may lead to activation of a corresponding subregion of the brain^78^, which may influence the CSF flow in that region. Therefore, further exploration using diverse stimulation parameters and targets would be necessary to fully understand the relationship between local neuronal activity and CSF solute transport.

The choice of an OA as a CSF tracer (M_W_ of 45 kDa) was based on its size similarity to the neuroactive Aβ oligomers, which are widely associated with Alzheimer's disease^79^. Although there was no direct evidence in the present study suggesting that neural activity improves waste clearance, it is possible that the wait time after the injection of the CSF tracer might have allowed the tracers to start exiting the brain^80^. While we have not examined the degree of tracer clearance to the cervical lymph nodes (primary sites of solute drainage from the brain), it is plausible to conjecture that neuronal activity may promote the solute clearance whereby prior research has established a functional relationship between a degree of CSF solute influx to brain parenchyma and its lymphatic drainage^81^. Thus, the effects of neuronal activity in the transport and clearance of different sizes of solutes in both CSF and interstitial space, including the assessment of solute drainage to cervical lymph nodes, will cast interesting subjects of future investigation.

## Conclusions

We presented early evidence of the neuronal activity elicited by electrical forelimb stimulation which increased regional CSF solute transport in the sensorimotor areas, with the inclusion of other brain areas that are in CSF transport pathways. Interhemispheric differences in CSF solute transport were observed in the stimulated somatosensory area, whereby the sustained stimulation without resting periods which reduced CSF transport efficiency may be associated with synaptic fatigue. The present work provides new insights to the link between neuronal activity and CSF solute transport. Further investigations are necessary to fully comprehend the scope of the relationship between neuronal activity and waste transport/clearance for the potential discovery of novel strategies for neurodegenerative disorders related to aberrant brain waste clearance.

### Supplementary Information

Supplementary Information.

## Data Availability

The data that support the findings of this study are available from the corresponding author upon reasonable request.
