# Supplementary material for: Cerebrospinal fluid solute transport associated with sensorimotor brain activity in rodents
Source: Sci Rep. 2023 Oct 9;13:17002. doi: 10.1038/s41598-023-43920-2 (PMC10562378; doi:10.1038/s41598-023-43920-2)
Supplement: Supplementary file 1 — Supplementary Information. [file 41598_2023_43920_MOESM1_ESM.pdf]

Cerebrospinal fluid solute transport associated with sensorimotor brain activity in rodents

Evgenii Kim[1], Jared Van Reet[1], Seung-Schik Yoo[1,*]

[1] Department of Radiology, Brigham and Women's Hospital, Harvard Medical School, Boston, MA

Supplementary materials

* All the correspondence to:

Seung-Schik Yoo, Ph.D.

Department of Radiology

Brigham and Women's Hospital

Harvard Medical School

75 Francis Street

Boston, MA 02115, USA

E-mail: yoo@bwh.harvard.edu

Table S1. The individual mean pixel intensity (MPI) values obtained from each region of interest (ROI) within the control (Ctrl) group. *p*-values were calculated by performing an interhemispheric comparison using a one-tailed, paired t-test. Brain regions that were not used for bilateral comparison are indicated as "N/A" in the *p*-value cells.

Total = total area of brain slices, MV = medial ventral region, MD = medial dorsal region, M1 = primary motor cortex, S1 = sensory cortex, TC = temporal cortex, OT = olfactory ventricle, VS = ventromedial striatum, and LV = lateral ventricle. CL and IL refer to hemispheres contralateral and ipsilateral to stimulated limb, respectively.

Animal ID	Ctrl														
	Total	MV	MD	M1		S1		TC		OT		VS		LV	
				CL	IL	CL	IL	CL	IL	CL	IL	CL	IL	CL	IL
C1	13.7	19.4	24.4	9.9	11.6	10.2	10.8	15.7	17.4	21.0	27.0	9.9	11.3	11.9	11.6
C2	18.1	26.7	37.5	14.0	11.2	14.5	12.6	19.1	22.2	25.3	30.4	14.2	12.0	18.5	21.7
C3	23.1	31.6	37.9	20.8	20.1	16.8	18.4	27.1	26.2	39.0	35.3	16.5	17.2	19.6	20.5
C4	28.2	39.5	45	22.1	18.1	24.3	18.6	45.1	31.5	53.7	44.8	18.9	16.9	24.9	24.7
C5	21.2	31.8	34.9	16.0	18.6	14.6	15.7	31.8	26.6	42.0	40.9	14.1	14.8	15.6	17.1
C6	20.3	31.6	33.4	18.4	17.5	16.4	13.7	27.8	23.7	33.8	34.6	14.3	13.8	16.7	15.9
C7	14.6	20.1	32.7	9.1	10.0	8.2	9.3	18.5	17.9	34.3	22.9	9.5	11.0	13.8	14.0
C8	18.5	25.8	37.4	13.9	15.7	12.4	15.7	23.5	25.8	32.7	37.2	10.1	11.4	15.1	15.0
C9	21.8	28.3	36.7	14.6	16.7	14.7	16.8	25.8	34.2	38.9	43.2	14.5	13.9	18.4	18.1
C10	10.0	10.7	17.7	9.2	7.2	9.3	7.3	12.5	10.6	17.0	14.9	9.1	8.1	9.1	9.7
mean	19.0	26.6	33.8	14.8	14.7	14.2	13.9	24.7	23.6	33.8	33.1	13.1	13.0	16.3	16.8
std	4.9	7.7	7.3	4.4	4.1	4.4	3.7	8.9	6.6	10.2	8.9	3.1	2.7	4.2	4.4
p-value	**N/A**	**N/A**	**N/A**	**0.425**		**0.380**		**0.285**		**0.369**		**0.419**		**0.1**	

Table S2. The individual mean pixel intensity (MPI) values obtained from each region of interest (ROI) within the intermittently (INT) stimulated group. *p*-values were calculated by performing an interhemispheric comparison using a one-tailed, paired t-test. *p*-values < 0.05 were highlighted in red. Brain regions that were not used for bilateral comparison are indicated as "N/A" in the *p*-value cells.

Animal ID	INT														
	Total	MV	MD	M1		S1		TC		OT		VS		LV	
				CL	IL	CL	IL	CL	IL	CL	IL	CL	IL	CL	IL
I1	31.6	52.8	46.3	27.4	23.2	28.7	24.5	43.2	35.7	58.3	46.2	21.6	19.3	32.0	27.7
I2	25.0	42.7	35.4	17.9	19.4	16.2	18.5	32.4	32.5	40.4	37.4	16.3	19.3	25.3	23.4
I3	27.4	51.8	38.7	22.9	13.5	26.5	13.9	45.6	23.3	52.2	33.0	20.1	15.9	32.0	26.3
I4	20.6	36.6	30.9	14.5	15.6	13.3	14.9	23.8	30.9	32.7	35.8	12.7	14.9	14.5	19.2
I5	23.6	37.7	27.9	17.9	14.7	17.0	14.4	30.9	26.5	48.4	39.0	18.1	16.4	31.7	29.6
I6	16.9	33.6	19.8	11.6	10.7	11.4	11.7	21.7	21.7	29.1	27.6	13.0	12.3	15.7	14.1
I7	23.8	36.4	31	18.6	13.8	19.4	13.8	30.1	29.8	36.7	33.5	16.8	16.1	27.0	28.9
I8	34.0	63.5	48.4	24.6	24.8	21.2	20.4	49.3	39.5	56.9	46.2	19.9	22.9	37.5	36.2
I9	21.1	41.5	30.5	19.7	15.0	16.9	13.3	32.7	23.8	48.2	28.6	14.9	12.5	19.1	15.2
I10	19.1	31.7	32	12.7	14.9	12.4	12.1	27.0	18.9	31.9	22.7	12.9	14.4	20.5	22.0
mean	24.3	42.8	34.1	18.8	16.6	18.3	15.8	33.7	28.3	43.5	35.0	16.6	16.4	25.5	24.3
std	5.1	9.6	8.1	4.9	4.3	5.5	3.9	8.9	6.2	10.2	7.2	3.1	3.1	7.4	6.5
p-value	**N/A**	**N/A**	**N/A**	**0.045**		**0.047**		**0.030**		**0.003**		**0.387**		**0.117**	

Table S3. The individual mean pixel intensity (MPI) values obtained from each region-of-interest (ROI) within the sequentially (SEQ) stimulated group. *p*-values were calculated by performing an interhemispheric comparison using a one-tailed, paired t-test. *p*-values < 0.05 were highlighted in red. Brain regions that were not used for bilateral comparison are indicated as "N/A" in the *p*-value cells.

Animal ID	SEQ															
	Total	MV	MD	M1		S1		TC		OT		VS		LV	
				CL	IL	CL	IL	CL	IL	CL	IL	CL	IL	CL	IL
S1	20.7	44.5	30.9	13.8	17.2	9	16.5	27	37.7	34.4	39.8	8.8	10.8	11	15.4
S2	22.7	46	33.3	13.5	17.2	12.7	17.6	18.7	30.6	23	34.1	15.9	20.1	29.2	29.6
S3	34.3	53.7	55	26.6	26.8	26.7	26	41.3	42.8	42.3	49.8	24.3	22.3	42.8	33
S4	33.1	70.3	45.6	26.5	20.6	23.6	19.8	52.6	42.3	59.7	56.4	23.9	20	26.8	24.7
S5	39.3	67.6	60.1	31.5	35.8	30.6	30.3	57.4	44.3	64	56.3	24.8	21.1	40.2	36.8
S6	26.9	56.1	32.3	15.3	19.1	15.1	18.7	34.6	44.2	41.8	48.6	18.3	20.1	21	22.8
S7	31.9	54.7	41.7	22.8	21	20.6	20.8	35.5	43.4	40	42.8	24.5	21.1	44.2	35.6
S8	31.3	63.9	46.7	23.3	32.1	19.2	28.5	33.2	38.8	41.8	47.3	18.6	24.4	24.5	30.7
S9	34.7	65.1	58.6	24.7	30.1	22	27.5	43.6	42.2	54.3	53	19.6	25.1	29.8	29.8
S10	32.3	65.1	38.1	21	22.8	20.9	22.3	35.9	40.2	46.9	54.2	24.1	21.1	37	34.6
mean	30.7	58.7	44.2	21.9	24.3	20	22.8	38	40.6	44.8	48.2	20.3	20.6	30.6	29.3
std	5.4	8.6	10.3	5.7	6.2	6.1	4.7	10.8	4	11.5	7	4.9	3.7	10	6.3
p-value	**N/A**	**N/A**	**N/A**	**0.049**		**0.031**		**0.178**		**0.047**		**0.399**		**0.788**	

Table S4. Group comparison of MPI values across brain regions using one-way ANOVA followed by Fisher's Least Significant Difference (LSD) *post-hoc* analysis. *p*-values < 0.05 were highlighted in red.

		Total	MV	MD	M1		S1		TC		OT		VS		LV	
					CL	IL	CL	IL	CL	IL	CL	IL	CL	IL	CL	IL
One-way ANOVA	F(2,27)	11.71	4.25	30.9	4.53	9.47	2.86	11.8	4.53	21.3	2.9	10.1	7.99	12.7	8.21	10.5
	p-value	< 0.01	0.03	< 0.01	0.02	< 0.01	0.07	< 0.01	0.02	< 0.01	0.07	< 0.01	< 0.01	< 0.01	< 0.01	< 0.01
LSD, *p*-value	Ctrl vs INT	0.04	0.94	< 0.01	0.10	0.43	0.11	0.34	0.06	0.10	0.06	0.61	0.06	0.03	0.02	0.01
	Ctrl vs SEQ	< 0.01	0.02	< 0.01	0.01	< 0.01	0.03	< 0.01	0.01	< 0.01	0.04	< 0.01	< 0.01	< 0.01	< 0.01	< 0.01
	SEQ vs INT	0.01	0.02	< 0.01	0.20	< 0.01	0.50	0.00	0.35	0.00	0.79	< 0.01	0.05	0.01	0.16	0.08

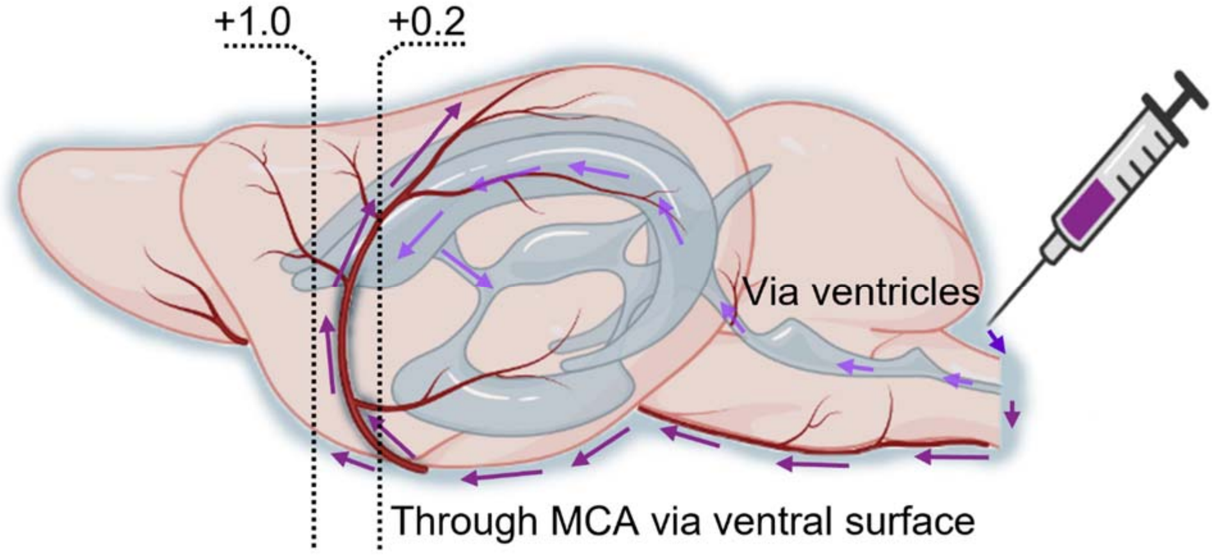

Supplementary Figure S1. Illustration of two prevailing pathways of intracisternal injected tracers. According to refs [1,2], two main routes - either (1) along the ventricular compartment (in purple arrows) or (2) along the ventral surface of the brain and the PVS abutting the middle cerebral arteries (MCA) (shown in burgundy arrows). Figure created with BioRender.com.

References

1. Benveniste H, Heerdt PM, Fontes M, Rothman DL, Volkow ND. Glymphatic system function in relation to anesthesia and sleep states. *Anesthesia & Analgesia* 2019; 128(4)**:** 747-758.

2. Gakuba C, Gaberel T, Goursaud S, Bourges J, Di Palma C, Quenault A *et al.* General anesthesia inhibits the activity of the “glymphatic system”. *Theranostics* 2018; 8(3)**:** 710.
